# Covert dissemination of carbapenemase-producing *Klebsiella pneumoniae* (KPC) in a successfully controlled outbreak: long- and short-read whole-genome sequencing demonstrate multiple genetic modes of transmission

**DOI:** 10.1093/jac/dkx264

**Published:** 2017-08-07

**Authors:** Jessica Martin, Hang T. T Phan, Jacqueline Findlay, Nicole Stoesser, Louise Pankhurst, Indre Navickaite, Nicola De Maio, David W Eyre, Giles Toogood, Nicolas M Orsi, Andrew Kirby, Nicola Young, Jane F Turton, Robert L. R Hill, Katie L Hopkins, Neil Woodford, Tim E. A Peto, A. Sarah Walker, Derrick W Crook, Mark H Wilcox

**Affiliations:** 1 Healthcare-Associated Infection Research Group, University of Leeds, Leeds, UK; 2 Clinical Microbiology Department, Leeds Teaching Hospitals NHS Trust, Leeds, UK; 3 Nuffield Department of Clinical Medicine, University of Oxford, Oxford, UK; 4 NIHR Health Protection Unit in Healthcare Associated Infections and Antimicrobial Resistance at University of Oxford in partnership with Public Health England, Oxford, UK; 5 Antimicrobial Resistance and Healthcare Associated Infections (AMRHAI) Reference Unit, National Infection Service, Public Health England, London, UK; 6 Department of Hepatobiliary Surgery, Leeds Teaching Hospitals NHS Trust, Leeds, UK

## Abstract

**Background:**

Carbapenemase-producing Enterobacteriaceae (CPE), including KPC-producing *Klebsiella pneumoniae* (KPC*-Kpn*), are an increasing threat to patient safety.

**Objectives:**

To use WGS to investigate the extent and complexity of carbapenemase gene dissemination in a controlled KPC outbreak.

**Materials and methods:**

Enterobacteriaceae with reduced ertapenem susceptibility recovered from rectal screening swabs/clinical samples, during a 3 month KPC outbreak (2013–14), were investigated for carbapenemase production, antimicrobial susceptibility, variable-number-tandem-repeat profile and WGS [short-read (Illumina), long-read (MinION)]. Short-read sequences were used for MLST and plasmid/Tn*4401* fingerprinting, and long-read sequence assemblies for plasmid identification. Phylogenetic analysis used IQTree followed by ClonalFrameML, and outbreak transmission dynamics were inferred using SCOTTI.

**Results:**

Twenty patients harboured KPC-positive isolates (6 infected, 14 colonized), and 23 distinct KPC-producing Enterobacteriaceae were identified. Four distinct KPC plasmids were characterized but of 20 KPC*-Kpn* (from six STs), 17 isolates shared a single pKpQIL-D2 KPC plasmid. All isolates had an identical transposon (Tn*4401*a), except one KPC*-Kpn* (ST661) with a single nucleotide variant. A sporadic case of KPC*-Kpn* (ST491) with Tn*4401*a-carrying pKpQIL-D2 plasmid was identified 10 months before the outbreak. This plasmid was later seen in two other species and other KPC*-Kpn* (ST14,ST661) including clonal spread of KPC-*Kpn* (ST661) from a symptomatic case to nine ward contacts.

**Conclusions:**

WGS of outbreak KPC isolates demonstrated *bla*_KPC_ dissemination via horizontal transposition (Tn*4401*a), plasmid spread (pKpQIL-D2) and clonal spread (*K. pneumoniae* ST661). Despite rapid outbreak control, considerable dissemination of *bla*_KPC_ still occurred among *K. pneumoniae* and other Enterobacteriaceae, emphasizing its high transmission potential and the need for enhanced control efforts.

## Introduction

Carbapenemase-producing Enterobacteriaceae (CPE) are an increasing threat to patient safety and the function of healthcare institutions in Europe and worldwide.^1^ Genes encoding carbapenemases may be integrated in the bacterial chromosome but are most often located on mobile elements, such as plasmids or transposons, which are transferable between bacterial strains, species and genera.^1^^,^^2^ Thus, clinical outbreaks are often complex, comprising varying degrees of clonal, plasmid or transposon-mediated gene spread. *Klebsiella pneumoniae* carbapenemases (KPC), encoded by *bla*_KPC_ alleles, have disseminated globally, primarily due to the clonal spread of KPC-producing *K. pneumoniae* (KPC*-Kpn*) strains, such as sequence type (ST) 258.^1^^,^^3^^,^^4^ However, in the last decade, dispersal of the transposable Tn*4401* element harbouring *bla*_KPC_^5–7^ amongst diverse plasmid structures, bacterial clones and species has increasingly been observed.^2^^,^^8^

KPC-*Kpn* have spread rapidly and extensively in some countries. In Italy, widespread nosocomial dissemination followed the introduction of KPC in 2008, leading to one-third of invasive *K. pneumoniae* isolates being carbapenem resistant in 2014.^9^ In the USA, KPC has spread to most states. In both countries, the outbreaks were predominantly due to transmission of KPC-*Kpn* ST258, and other clonal group 258 STs, such as ST512.^1^^,^^10^

KPC was first recognized in the UK in 2003.^11^ Since then, the incidence of KPC-producing Enterobacteriaceae (KPC-E) has risen,^12^ with 664 isolates referred to PHE’s Antimicrobial Resistance and Healthcare Associated Infections (AMRHAI) Reference Unit in 2015.^13^ Twenty-three *bla*_KPC_ variants have been described, but most UK isolates carry either *bla*_KPC-2_, *bla*_KPC-3_ or occasionally *bla*_KPC-4_.^14^ More than 95% of UK KPC-E originate from northwest England where transmission has principally occurred horizontally via variants of an epidemic plasmid (pKpQIL).^12^^,^^14^ When seen outside northwest England, previous UK *bla*_KPC_ isolates have been typically associated with *K. pneumoniae* clonal group 258.^12^^,^^14^^,^^15^

Whole-genome sequencing (WGS) is a highly discriminatory typing technique used to investigate pathogen transmission in nosocomial outbreaks. Genetic relatedness amongst strains is determined by nucleotide-level variation.^16–18^ However, analysing plasmid structures using short-read sequencing remains challenging,^19^ limiting the complete elucidation of *bla*_KPC_ transmission networks. Recently, data from long-read platforms, particularly PacBio’s single molecule real-time (SMRT) sequencers, have enabled plasmid-level analyses, in some instances leading to an improved understanding of horizontal *bla*_KPC_ spread.^8^^,^^20^ Oxford Nanopore Technology’s (ONT) long-read MinION sequencers represent a potentially quicker and more cost-effective alternative.

In 2013, Leeds Teaching Hospitals NHS Trust (LTH), UK, experienced a short-lived poly-species KPC-E outbreak on a single hospital ward. We investigated the molecular epidemiology of this temporo-spatially limited outbreak using WGS and detailed hospital admissions data to investigate the likely transmission mechanisms. The characterization of *bla*_KPC_ plasmid structures was facilitated by MinION sequencing data, marking the first application of MinION long-read sequencing technology to tracking *bla*_KPC_ plasmid diversity in a clinical outbreak. We also attempted to elucidate the infection prevention and control interventions likely to have brought the outbreak under control.

## Materials and methods

### Setting

LTH is a large tertiary referral centre (>2000 beds), including a 32-bed supra-regional liver unit with eight single rooms, and six 4-bed bays. Patients are regularly referred from outside the region (including northwest England) for liver transplantation. The LTH liver unit experienced only rare, sporadic cases of infection/colonization with KPC-E before 2013 (one case >18 months prior to outbreak), with no previous evidence of patient-to-patient CPE transmission. Admission CPE screening using rectal swabs was commenced for all LTH liver unit admissions in 2011 following the introduction of UK CPE guidance.^21^

### KPC-E outbreak and case definitions

An outbreak was declared in October 2013 following reference laboratory confirmation of three LTH liver unit patients carrying KPC*-Kpn* with the same variable-number-tandem-repeat (VNTR)^22^^,^^23^ profile (6,4,2,0,2,2,2,3,1). The initial outbreak definition included patients with KPC-*Kpn* with this VNTR type, as it was rare in the UK (19/922 national isolates; J. F. Turton AMRHAI unpublished data, 2012–13). However, this definition was almost immediately broadened to include all KPC-E once plasmid dispersal was suspected.

Clinical cases were defined as patients with KPC-E isolated from a clinical sample who had signs and symptoms consistent with infection. Colonized cases were defined as patients who had KPC-E identified on rectal screening without clinical evidence of infection. Details on ward movements, sampling and clinical outcomes were collected retrospectively from electronic healthcare records and clinical notes.

### Infection prevention and control interventions

A multifaceted infection prevention and control strategy was immediately implemented when the outbreak was detected and continued until the outbreak was declared over (January 2014). The strategy targeted five main areas: hand hygiene, cohorting and isolation, screening, environmental cleaning, and education (Table S1, available as Supplementary data at *JAC* Online). Interventions were regularly reviewed in multidisciplinary outbreak team meetings. The outbreak was limited to patients on a single ward and interventions were targeted to this location.

### CPE screening including microbiology methods

At the outbreak onset, enhanced rectal screening was introduced, i.e. for all patients on admission and discharge from the liver unit (including to/from other wards), and weekly following admission. Rectal CPE screening swabs were cultured using ESBL chromogenic agar with a 10 μg ertapenem disc (Oxoid, Basingstoke, UK) since KPC producers are typically resistant to cephalosporins. Species identification (using MALDI-TOF) was performed for all Enterobacteriaceae from clinical samples and screening swabs with reduced ertapenem susceptibility (zone diameter <26 mm, by EUCAST disc testing^24^); isolates were phenotypically investigated for carbapenem resistance using the Modified Hodge test, Rosco discs (KPC/Metallo-B-Lactamase Confirm kit, Rosco, Denmark) and by determining ertapenem MICs (Etest, bioMérieux, UK). All potential CPE-producing isolates were sent to the AMRHAI Reference Unit for PCR investigation for *bla*_OXA-48_, *bla*_IMP_, *bla*_NDM_, *bla*_VIM_ and *bla*_KPC._^25–28^ Plasmids were typed using a specifically designed pKpQIL-like plasmid PCR^29^ targeting six signature sites.

### DNA extraction and sequencing

DNA was extracted from a single colony after subculture of frozen KPC-E isolates using a commercial kit (Quickgene, Fujifilm) as per the manufacturer’s instructions, with an additional mechanical lysis step following chemical lysis (FastPrep, MP Biomedicals). WGS used Illumina Hiseq 2500 sequencing technology, with a sequencing depth of ∼100 × per sample. We selected five isolates for MinION sequencing: one random dominant strain KPC-*Kpn-*ST661 isolate and four with *bla*_KPC_ plasmid structures that were thought to be distinct (short-read WGS-based analysis, three patients). Genomic DNA was isolated using the Qiagen Genomic-tip 100/G kit (Qiagen, Germany) following the manufacturer’s recommendations. Isolates were prepared for 2D sequencing using the SQK-NSK007 sequencing kit with no DNA shearing and modifications to minimize DNA fragmentation and preserve plasmid structures (see Supplementary data: Methods).

### Sequence processing and analysis

Species identification was performed on short-read Illumina data using Kraken^30^ with a reduced database comprising human, bacterial and viral genomes. Reads were then mapped to species-specific reference sequences and base calling was performed as previously described.^31^ Resistance mechanisms were identified using resistType (https://github.com/hangphan/resistType), a method combining both assembly/BLASTn and mapping of sequencing reads to a panel of known reference mechanisms, including both chromosomal and acquired mechanisms.

Consensus FASTA sequences from the pipeline were used to reconstruct phylogenetic trees for each species with IQTree,^32^ using a GTR + G model and a maximum parsimony starting tree. The phylogeny was corrected for recombination using ClonalFrameML with default parameters.^33^ Short-read sequences were assembled using SPAdes^34^ (version 3.6) and the assemblies used for *in silico* multilocus sequence-typing (MLST), plasmid Inc typing and Tn*4401* typing by BLASTn. For plasmid typing, all publicly available complete plasmids were downloaded from NCBI (query term: plasmids AND Enterobacteriaceae AND complete sequence), deduplicated, and plasmids carrying *bla*_KPC_ alleles were extracted. Additional fully closed plasmid sequences were obtained from a global KPC study.^35^ These plasmid sequences were then used as references to identify similar *bla*_KPC_-carrying plasmid structures in the outbreak using BLASTn.

MinION sequencing data were processed by poretools^36^ to extract 2D reads. Multiple approaches to generate assemblies were applied using both MinION long-read and Illumina short-read data, namely hybridSPAdes,^34^ npScarf^37^ and Canu^38^ (Supplementary data: Methods). A plasmid was defined as circularized if it had >100 bp overlapping ends with 100% sequence identity for hybridSPAdes/npScarf assemblies, and >1 kbp overlapping ends at >99% sequence identity for Canu assemblies.

We applied SCOTTI,^39^ a structured coalescent-based tool for reconstructing transmission within outbreaks, to the dominant outbreak KPC-*Kpn* ST661 isolates, combining epidemiological and chromosomal genomic data. We masked the recombinant regions detected by ClonalFrameML, and used the resulting genome alignment as input to SCOTTI, together with the first date where a KPC-E isolate was detected in a patient, and the start and end date of each patient’s infection risk period (Supplementary data: Methods). We used a π prior distribution for the mutation rate (mean 2 × 10^–^^6^)^31^ and a uniform prior distribution between 14 and 16 for the number of hosts (also allowing possible non-sampled hosts).

All short-read and long-read WGS data are available in the NCBI repository under project number PRJNA353334.

## Results

Twenty identified patients (6 cases, 14 colonized; Table 1) were included in the outbreak analysis; 17 were detected in LTH during the outbreak period, 2 cases were identified after transfer from LTH to other UK hospitals and 1 patient was identified (by the AMRHAI Reference Unit) as a potential index case due to it sharing a KPC-*Kpn*-associated pKpQIL-D2 variant plasmid^29^ (December 2012; case 1). The first KPC-E of each species for each patient was analysed, giving rise to 23 distinct KPC-E isolates (all *bla*_KPC_-positive) including: 20 *K. pneumoniae* (6 distinct STs; 15 ST661 and 5 STs with a single isolate), 1 *Citrobacter freundii*, 1 *Klebsiella oxytoca* and 1 *Enterobacter cloacae*. Two patients (cases 6 and 12) had two distinct KPC-E and one patient (case 2) had two *K. pneumoniae* isolates (with differing antimicrobial susceptibility profiles) analysed.

**Table 1.** Summary of cases and isolates involved in the LTH KPC outbreak

Case	First KPC positive sample	Sample type	Clinical status	Isolate species	VNTR type	Sequence type	Plasmid type	Replicon type	Tn*4401* type	Clinical outcome
1	13/12/12	rectal screen	colonized	*K. pneumoniae*	8,5,4,2,1,1,3,2,1	Kpne-ST-491	pKpQIL-D2	IncFIB(K),IncFIB(pKPHS1), IncHI2,IncHI2A,IncR	Tn*4401*a	recovered
2	10/07/13	rectal screen	colonized	*K. pneumoniae*	6,4,-,0,-,2,2,3,1	Kpne-ST-661	pKpQIL-D2	IncFIB(K),IncFII,IncFII,IncR	Tn*4401*a	recovered
2	10/07/13	rectal screen	colonized	*K. pneumoniae*	6,4,-,0,-,2,2,3,1	Kpne-ST-661	pKpQIL-D2	Col(MG828),Col156,IncFIB(K), IncFII,IncFII,IncR	Tn*4401*a	recovered
3	13/07/13	rectal screen	colonized	*K. pneumoniae*	6,4,-,0,-,2,2,3,1	Kpne-ST-661	pKpQIL-D2	IncFIB(K),IncR	Tn*4401*a	recovered
4	03/10/13	intra-operative swab	case	*K. pneumoniae*	6,4,2,0,-,2,2,3,1	Kpne-ST-661	pKpQIL-D2	IncFIB(K),IncR	Tn*4401*a	died <30 days
5	09/10/13	rectal screen	colonized	*K. pneumoniae*	6,4,-,0,-,2,2,3,1	Kpne-ST-661	pKpQIL-D2	IncFIB(K),IncR	Tn*4401*a	recovered
6	13/10/13	sputum	colonized	*E. cloacae*	NA	Eclo-ST-144	pKpQIL-D2	IncFIB(K),IncR	Tn*4401*a	recovered
6	13/10/13	sputum	colonized	*K. pneumoniae*	NA	Kpne-ST-661	pKpQIL-D2	IncFIB(pECLA),IncR	Tn*4401*a	–
7	17/10/13	sputum	colonized	*K. pneumoniae*	6,4,2,0,2,2,2,3,1	Kpne-ST-661	pKpQIL-D2	IncFIB(K),IncR	Tn*4401*a	recovered
8	20/10/13	mid-stream urine	case	*K. pneumoniae*	6,4,2,0,2,2,2,3,1	Kpne-ST-661	pKpQIL-D2	IncFIB(K),IncR	Tn*4401*a	died <30 days
9	25/10/13	rectal screen	colonized	*K. pneumoniae*	6,4,2,0,2,2,2,3,1	Kpne-ST-661	pKpQIL-D2	IncFIB(K),IncR	Tn*4401*a [986G>T]	recovered
10	25/10/13	rectal screen	colonized	*K. oxytoca*	NA	Koxy-NF	pKpQIL-D2	IncFIB(K),IncR	Tn*4401*a	recovered
11	01/11/13	rectal screen	colonized	*K. pneumoniae*	6,4,2,0,2,2,2,3,1	Kpne-ST-661	pKpQIL-D2	Col(IMGS31),IncN,IncR	Tn*4401*a	recovered
12	04/11/13	rectal screen	colonized	*C. freundii*	NA	Cfre-NF	distinct	IncFIA(HI1),IncFIB(K), IncFII(Yp),IncN,IncR	Tn*4401*a	recovered
12	04/11/13	rectal screen	colonized	*K. pneumoniae*	NA	Kpne-ST-250	distinct	IncFIB(K),IncR	Tn*4401*a	–
13	08/11/13	rectal screen	colonized	*K. pneumoniae*	distinct	Kpne-ST-14	pKpQIL-D2	IncFIB(K),IncR	Tn*4401*a	recovered
14	19/11/13	rectal screen	colonized	*K. pneumoniae*	6,4,2,0,2,2,2,3,1	Kpne-ST-661	pKpQIL-D2	IncFIB(K),IncR	Tn*4401*a	recovered
15	25/11/13	blood culture	case	*K. pneumoniae*	6,4,2,0,2,2,2,3,1	Kpne-ST-661	pKpQIL-D2	IncFIB(K),IncR	Tn*4401*a	died <30 days
16	19/12/13	rectal screen	colonized	*K. pneumoniae*	distinct	Kpne-ST-1820	distinct	IncFIB(K)	Tn*4401*a	recovered
17	07/01/14	rectal screen	colonized	*K. pneumoniae*	6,4,2,0,2,2,2,3,1	Kpne-ST-661	pKpQIL-D2	IncFIB(K),IncFII(K),IncR	Tn*4401*a	recovered
18	08/01/14	rectal screen	colonized	*K. pneumoniae*	distinct	Kpne-ST-321	pKpQIL-D2 like	IncFIB(K),IncFIB(pKPHS1),IncR	Tn*4401*a	recovered
19	16/01/14	rectal screen	colonized	*K. pneumoniae*	6,4,2,0,2,2,2,3,1	Kpne-ST-661	pKpQIL-D2	IncFIB(K),IncR	Tn*4401*a	recovered
20	20/01/14	mid-stream urine	case	*K. pneumoniae*	6,4,2,0,2,2,2,3,1	Kpne-ST-661	pKpQIL-D2	IncFIB(K),IncR	Tn*4401*a	recovered

VNTR, variable-number-tandem repeat; NA, not available.

**Figure 1.**
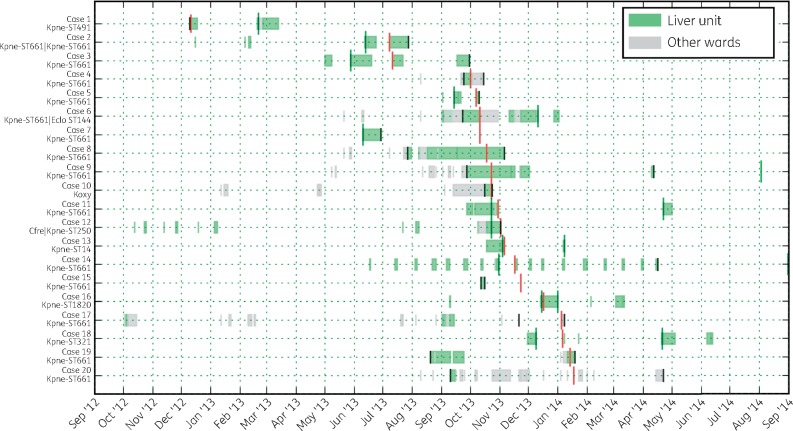
Patient admissions to LTH liver unit and other LTH wards during the outbreak period. Red vertical marks are the time of first KPC detection in the patients, black vertical marks denote the starts and ends of the infection risk periods defined for SCOTTI, green vertical marks denote the closest KPC-negative screening time points (where applicable) before or after the first KPC-positive screening result. This figure appears in colour in the online version of *JAC* and in black and white in the print version of *JAC*.

The median patient age was 56 years (range 32–76), and 12/20 (60%) patients were male. 8/20 (40%) patients were liver transplant recipients. Most (19/20) cases (cases 2–20) spent time on the LTH liver unit between July 2013 and January 2014 and were presumed nosocomial acquisitions (Figure 1). *bla*_KPC_ was detected >48 h post-admission for 9 cases. For the remaining 10 patients (excluding the index case), typically with short-lived but regular admissions to the unit, there was a median 25 days (range 10–125, IQR 16–73) between the most recent admission to the unit and first *bla*_KPC_ detection. Six patients experienced clinical infection associated with a KPC-producing isolate July 2013–January 2014; three had KPC-E bacteraemia, and one each had urinary tract, lower respiratory tract or surgical wound infections. The 30 day all-cause mortality among KPC-affected patients was 3/20 (15%).

The outbreak was deemed successfully controlled due to the lack of any new KPC-E colonization episodes/cases in LTH over the subsequent 21 months. The KPC-positive patients were not formally followed up after discharge as many were resident outside of Leeds. Of the 17 surviving discharged KPC-positive patients, 3/6 opportunistically screened ≥3 months post-diagnosis remained KPC-positive and 2/3 screened ≥6 months post-diagnosis remained positive. One patient remained KPC-positive 511 days post-diagnosis. One patient had three negative rectal swabs over a 6 month period, suggestive of *bla*_KPC_ clearance;^21^ the remainder had insufficient local screens to confirm/refute loss of carriage.

### WGS demonstrates multiple modes of transmission

**Figure 2.**
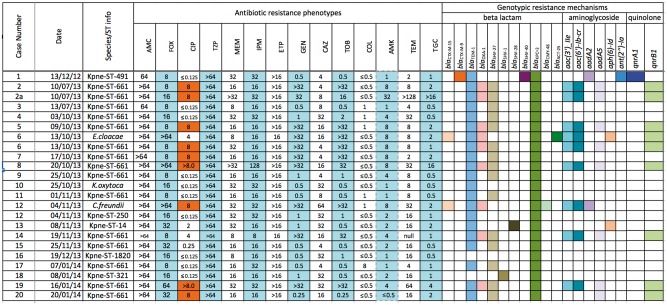
Phenotypic sensitivity and antimicrobial resistance gene prediction results of 23 strains from LTH KPC outbreak (MICs: mg/L measured by agar dilution at AMRHAI). AMC, co-amoxiclav; FOX, cefoxitin; CIP, ciprofloxacin; TZP, piperacillin/tazobactam; MEM, meropenem; IPM, imipenem; ETP, ertapenem; GEN, gentamicin; CAZ, ceftazidime; TOB, tobramycin; COL, colistin; AMK, amikacin; TEM, temocillin; TGC, tigecycline. This figure appears in colour in the online version of *JAC* and in black and white in the print version of *JAC*.

Sequence data were available for all (*n *=* *23) isolates (Figure 2). Twenty-two of 23 isolates had an identical transposon (Tn*4401*a,^5^ as in pIT-01C03, GenBank accession: HG969995.1), and one had just one single nucleotide variant (SNV) resulting in transposon type Tn*4401*a[986G > T]. This mutation results in a stop codon at position 261 in the resolvase/phage integrase protein facilitating site-specific recombination which could affect Tn*4401* mobility.

Variable phenotypic susceptibilities and antimicrobial resistance genotypes were noted amongst study isolates (Figure 2). All isolates carried *bla*_KPC-2_; no other carbapenem resistance mechanisms were demonstrated. Many also carried the ESBL gene *bla*_SHV-27_ (*n *=* *15) and the β-lactamase genes *bla*_TEM-1_ (*n *=* *21) and Δ*bla*_OXA-9_ (*n *=* *21). Other broad or extended-spectrum β-lactamase genes observed included *bla*_OXA-1_ (*n *=* *8), *bla*_CTX-M-1_ (*n *=* *2) *bla*_CTX-M-9_ (*n *=* *1) and *bla*_CMY-48_ (*n *=* *1). Some isolates also carried aminoglycoside resistance genes. The ciprofloxacin-resistant phenotype in 12 isolates (>0.5 mg/L) was inconsistently explained by relevant genotypes. High-level ciprofloxacin resistance (≥8.0 mg/L) was seen in nine isolates of which seven carried *qnrB1*. For the remainder, chromosomal *gyrA* mutations (S83Y/F) and uncharacterized changes in efflux pump activity might have contributed to resistance. Colistin resistance (8 mg/L) was observed in one isolate (case 17). WGS data of this isolate revealed the presence of neither *mcr-1* nor *mcr-2* but N42D amino acid change was observed in the chromosomal gene *mgrB* whose alterations (e.g. gene disruption by insertion sequences, missense or nonsense point mutations, and small deletions) can confer colistin resistance.^40^ The N42D mutation has not, however, to our knowledge been previously described as causative.

The WGS-based plasmid typing results mostly agreed with PCR-based typing (19/20 PCR-positive cases). Nineteen isolates yielded assemblies with contigs highly similar to plasmid pKpQIL-D2^29^ (>95% sequence length match/>99% sequence identity). pKpQIL-D2 is an IncR/IncFII *bla*_KPC-2_ plasmid, differing from pKpQIL in the partitioning and replication regions, without the pKpQIL signature replicon IncFIB(pQIL), and also harbouring *bla*_TEM-1_ and Δ*bla*_OXA-9_. pKPC18_LTH from case 18 (Kpne-ST321) was identified as pKpQIL-D2 by PCR but did not meet our predefined WGS-based plasmid typing thresholds for a match. However, on more detailed review, this isolate’s assembly included contigs matching 91% of pKpQIL-D2 at 99% sequence identity, and would be consistent with it being classified as a pKpQIL-D2-like plasmid affected by large indels (see long read results below). The remaining three isolates had distinct plasmid structures with significant matches to the NCBI plasmid database (threshold: sequence length match >75%; sequence identity >95%). Most isolates carried plasmid replicon types IncR (22/23) and IncFIB (22/23).

### bla_KPC_/Tn4401 plasmid characterization from MinION long-read sequencing

**Figure 3.**
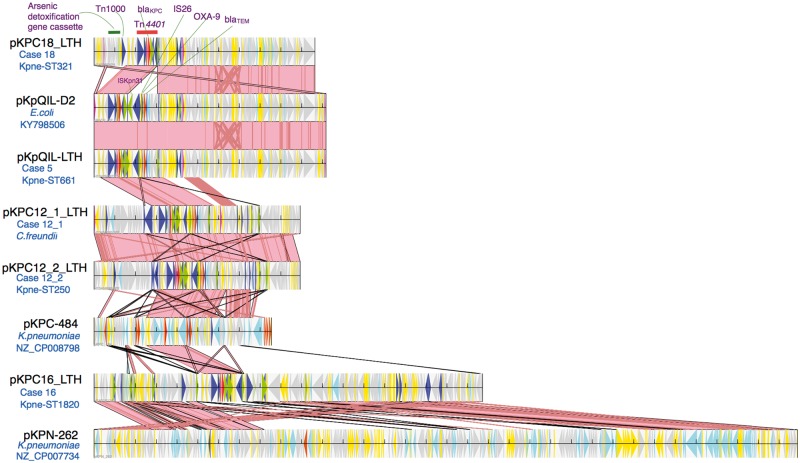
Outbreak KPC plasmids and contigs derived from long-read sequencing of isolates and their alignment to the most closely genetically matched complete plasmid sequences from NCBI. This figure appears in colour in the online version of *JAC* and in black and white in the print version of *JAC*.

MinION sequencing coverage for the five selected isolates ranged from 26× to 43× (Table S2), enough to facilitate complete assembly of the *bla*_KPC_/Tn*4401*-carrying plasmids in 4/5 isolates, either by hybridSPAdes (*n *=* *1) or Canu (*n *=* *3, Table S3). The closed structure of the *bla*_KPC_/Tn*4401*-carrying plasmid of the KPC*-Kpn* ST661 isolates, pKpQIL-LTH (117 kb), was 99% identical to the IncR/IncFII pKpQIL-D2 (Figure 3). The *bla*_KPC_/Tn*4401*-carrying plasmids from *C. freundii* (pKPC12_1_LTH, 99.3 kb) and KPC*-Kpn* ST250 (pKPC12_2_LTH, 99.3 kb) from case 12 showed nearly identical plasmid structure (>99% sequence length match and sequence identity; Figure 3). This was a novel *bla*_KPC_*-*harbouring IncN/IncR plasmid, most closely related to pKPC-484 (GenBank accession: CP008798.1, 85.5 kb, Maryland, USA; 56% sequence length match, 99% sequence identity). The distinct structure of this plasmid compared with the dominant outbreak pKpQIL-D2 plasmid (43% sequence length match including regions flanking Tn*4401*, 99% sequence identity) implicates either independent import of this plasmid from an unknown source, or a novel local transfer event of *bla*_KPC_/Tn*4401* into a new plasmid backbone, likely via recombination. The presence of these near-identical plasmids in two different species suggests cross-species transmission of this novel plasmid structure within this patient.

The *bla*_KPC_/Tn*4401* plasmid pKPC18_LTH from case 18 (KPC*-Kpn* ST321, Canu assembly, 106 kb) was an IncR/IncFII pKpQIL-D2-like plasmid (91% sequence length match, 99% sequence identity; Figure 3). It differed from pKpQIL-D2 by the deletion of the Δ*bla*_OXA-9_–*bla*_TEM-1_ gene cassette and the insertion of an arsenic detoxification gene cassette into the pKpQIL-D2 plasmid backbone. This patient from northwest England was admitted to LTH in December 2013, and was first found to be colonized with a KPC-positive isolate in January 2014. Therefore, this patient might have carried this plasmid prior to admission to LTH or acquired it locally from an unidentified source with large indel events potentially driven by selection pressures.

In the remaining case with a distinct plasmid (KPC*-Kpn* ST1820, case 16), manual inspection of the hybridSPAdes assembly implicated that there was an assembly error, incorrectly merging the *bla*_KPC_/Tn*4401* plasmid contig with the chromosomal contig (Figure S1). After error correction, the revised *bla*_KPC_/Tn*4401* contig pKPC16_LTH (187 kb) was 83% similar to pKPN-262 (Figure 3) harbouring the pKpQIL-like IncFII replicon and IncFIB(K) as opposed to IncFIB(pQil) generally found in pKpQIL-like plasmids. The patient harbouring pKPC16_LTH was admitted for liver transplant in December 2013 from Manchester and had negative screening results for *bla*_KPC_ on admission. This could imply either a horizontal transfer of the *bla*_KPC_/Tn*4401* element from pKpQIL-D2 outbreak plasmid into another plasmid backbone or an independent acquisition from a local unknown source.

### Transmission analysis

**Figure 4.**
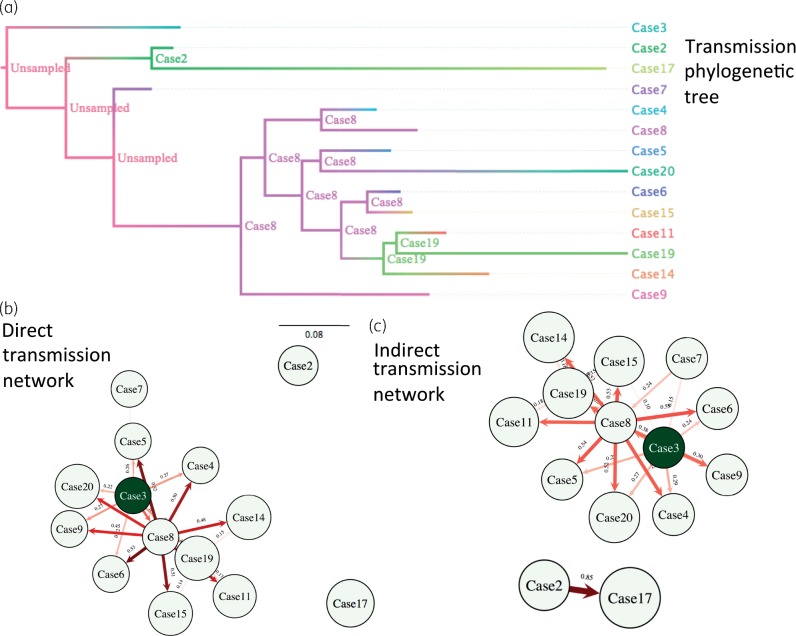
Transmission analysis results inferred by SCOTTI for the dominant outbreak strain KPC*-Kpne* ST661 using consensus chromosomal sequences and epidemiological data. The ‘maximum clade credibility’ of the posterior coalescent tree (scale in years) was inferred by SCOTTI, taking into account epidemiological data and transmission processes. This figure appears in colour in the online version of *JAC* and in black and white in the print version of *JAC*.

WGS-based chromosomal sequences and detailed epidemiological data were used to estimate probable *bla*_KPC_ transmission routes, with the aim of differentiating between ward-based dispersal and importation from high-risk areas outside, but associated with, Leeds. A potential index case of KPC*-Kpn* (ST491) with the pKpQIL-D2 Tn*4401*a-carrying plasmid was identified 10 months before the outbreak. This plasmid was later seen in other KPC*-Kpn* (ST14, ST661) and two other species, consistent with unrecognized plasmid dispersal, and subsequently a strain–plasmid (*K. pneumoniae* ST661-pKpQIL-LTH, Figure S2) outbreak nearly a year later. The transmission network and transmission phylogenetic tree (Figure 4) suggested that a single case (case 8) was potentially central to dissemination of ST661-pKpQIL-LTH following probable acquisition from case 3. This symptomatic case was in a shared bay for a prolonged period prior to KPC detection (Figure 1), potentially transmitting to nine cases (with >0.4 probability), of which six were within 2–4 SNPs (cases 4–7, 11 and 15). Patient-to-patient transmission was not demonstrated between other cases in the outbreak, for which KPC acquisition was more likely to relate to unidentified sources, such as unscreened patients, staff or environmental reservoirs. Cases 2 and 17 (7 SNPs apart) plausibly represented an indirect transmission link, probably through multiple transmission chains. Although case 17 was >10 SNPs different from other clonal strains, case 2 was 4–11 SNPs apart from the remaining cases. Due to large differences in hospital stays and times from colonization to sampling, coalescent times within hosts vary, giving rise to a broad range of SNP distances, and therefore absolute SNP distance thresholds are not informative and often bias to discriminate direct transmission links.

## Discussion

This is the first report of the detailed molecular epidemiology of a successfully controlled KPC-E outbreak in the UK. This study also marks the first application of MinION long-read sequencing to investigate *bla*_KPC_ plasmid diversity in a clinical outbreak. This nosocomial outbreak, involving 23 isolates from just 20 patients, demonstrated *bla*_KPC_ dissemination via multiple routes, including clonal spread of a bacterial strain in conjunction with a pKpQIL-D2 plasmid (*K. pneumoniae* ST661, 15 cases), horizontal transfer of the outbreak pKpQIL-D2 plasmid to other STs/species (5 cases) most likely following its introduction via an index case 10 months previously, and either horizontal transposition (Tn*4401*a transmission from the outbreak pKpQIL-D2 plasmid to other distinct plasmids) or external import of *bla*_KPC_/Tn*4401* plasmids. This small outbreak included striking genetic diversity (six *K. pneumoniae* lineages, cross-species plasmid transmission within patients, four distinct plasmid types) and highlights the relative impact of symptomatic patients, non-*K. pneumoniae* KPC-E, environmental reservoirs and unscreened individuals in *bla*_KPC_ dissemination (despite high-density contact-screening). The initial outbreak definition capturing only KPC-*Kpn* with the same VNTR type failed to demonstrate the extent of *bla*_KPC_ transmission, and reaffirms the need for consideration of multispecies involvement early in putative outbreaks. Patient-to-patient KPC-E transmission outside the liver unit could not be excluded but was thought unlikely as no new cases/colonized patients were detected outside the liver unit during the outbreak, or subsequently.

This outbreak demonstrates the difficulty in identifying transmission events between CPE cases, even with the aid of high-intensity screening, short-read WGS data and long-read plasmid sequencing. We demonstrated a high colonized : infected patient ratio (3:1) compared with other outbreaks,^41^ possibly reflecting more intensive screening or a lower rate of clinical infection in colonized individuals. Our analysis demonstrates probable dissemination of ST661-pKpQIL-D2 from a central source to nine cases, and indirect transmission for the remainder, supporting the existence of alternative unscreened reservoirs. Long-read MinION sequencing proved to be beneficial for high-resolution plasmid tracking, but still suffered from sequencing and bioinformatics limitations, resulting in an erroneous result in one assembly. Improvements in sequencing protocols to enrich for longer reads, better bioinformatics tools (assemblers) independent of highly skilled bioinformatics support, and cost reductions are required to implement routine WGS-based plasmid tracking. Additionally, although same-day, direct from sample, turnaround time as demonstrated for *Mycobacterium tuberculosis* diagnosis^42^ has not yet been achieved for Enterobacteriaceae species, current developments in culture-based laboratory protocols allow sample preparation for long-read sequencing to be completed in ∼8 h (6 h DNA extraction; 10 min or 1.5 h options for sequence library preparation; ONT). Subsequently, 80% of sequencing data can be obtained within the first 10 h of a 48 h sequencing run,^42^ and bioinformatics analysis can be performed in 4–5 h. This allows potential turnaround times of <24 h from culture positivity to initial outbreak analysis.

Nosocomial CPE outbreaks may evolve into regional endemicity if transmission chains between patients/other reservoirs cannot be interrupted. Patient transfer from high-prevalence KPC-E centres in northwest England to the Leeds supra-regional liver unit were frequent during this outbreak, representing multiple opportunities for reintroductions. A recent study suggests the most probable source of CPE is from readmission of patients from the same hospital/region rather than transfer between regions.^15^ Despite detailed genetic analysis it was difficult to differentiate clearly between importation of resistance vectors and patient-to-patient KPC-E transmission in this outbreak. However, the rare plasmid-type, dissemination of a rare lineage (ST661) and the fact that the outbreak was successfully controlled suggests local transmission was the dominant source of acquisition. Given that all patients on the unit were screened following detection of the outbreak, undetected human reservoirs were a less likely source of KPC acquisition than environmental persistence. Hydrogen peroxide vapour treatment of the unit on two occasions, along with strict patient cohorting and screening, may have contributed significantly to halting the outbreak.

This outbreak resulted in changes to clinical care: transplant assessments were transferred to the outpatient setting and included CPE screening, and strict algorithms for admission screening/isolation on the liver unit, discharge CPE screening and carbapenem-sparing antibiotic prescribing strategies were implemented. It was not possible to evaluate the relative impact of each of these interventions on reducing CPE transmission.

There were several limitations of this study, mostly relating to the potential to have underestimated cases/colonization episodes. Phenotypic tests were used to identify potential CPE producers during the outbreak; since then, more sensitive and specific PCR-based CPE detection has been implemented.^43^^,^^44^ There was a lack of high-intensity screening of asymptomatic patients prior to outbreak detection (July–October 2013). However, routine CPE admission screening was in place during this period. Repeat KPC-E isolates from individual patients were not available. Analysis of the evolution of KPC-E strains within individuals over time may have provided further information about the genetic mechanisms associated with *bla*_KPC_ dispersal. Finally, there are few publically available UK KPC-carrying whole-genome and plasmid sequences, limiting our ability to contextualize the Leeds strains.

We emphasize that there is considerable potential for rapid dissemination of carbapenemase genes among *Klebsiella* spp. and between Enterobacteriaceae, despite the implementation of extensive infection control interventions. Tracking transmission networks is challenging, even with detailed epidemiological and WGS data, due to the mobility and evolution of mobile genetic elements. Publicly available national CPE sequence data will enable local institutions to contextualize outbreak strains and identify the role of importation versus patient-to-patient transmission in response to increases in CPE incidence and outbreaks.

## Supplementary Material

Supplementary Data
