# Supplementary material for: Covert dissemination of carbapenemase-producing Klebsiella pneumoniae (KPC) in a successfully controlled outbreak: long- and short-read whole-genome sequencing demonstrate multiple genetic modes of transmission
Source: J Antimicrob Chemother. 2017 Aug 7;72(11):3025–34. doi: 10.1093/jac/dkx264 (PMC5890743; doi:10.1093/jac/dkx264)
Supplement: Supplementary Data [file dkx264_supplementary_data.docx]

## Supplementary data

**Methods**

***Culture and identification of carbapenem resistance***

Screening samples (Day 1, stage 2 workflow used for suspected carbapenem resistant isolates from clinical samples).

Day 0

1. Samples were direct plated onto ESBL chromogenic agar (Oxoid, Basingstoke, UK).
2. Samples were streaked to single colonies and a 10µ ertapenem disc (Oxoid, Basingstoke, UK) was placed in the area of the second streak
3. Plates were incubated in air at 35-37^o^ for 18-24 hours

Day 1

1. Ertapenem zone size was measured:
   1. If >28mm, oxidase testing was performed. If oxidase negative, for identification, sensitivity testing and ESBL and AMPC detection testing (D68C, Mast Group, UK)
   2. If <28mm, workflow detailed in 2.
   3. If zone too light to read, obscured or borderline, day 0 protocol was repeated.
2. For isolates with Ertapenem zone size <28mm:

Oxidase testing

Negative

Positive

Identification (Vitek) and extended sensitivity testing, including meropenem, ertapenem and imipenem. ESBL and AMPC detection testing (D68C, Mast Group, UK).

No further work

Negative

Enterobacteriaceae with confirmed carbapenem resistance were set up for modified-hodge testing and KPC/metallo-beta lactamase/OXA-48 disc testing (Rosco, Taastrup, Denmark).

All non-enterobacteriaceae had no further work if sensitivity pattern appropriate for species. If not typical, samples were discussed individually with duty microbiologist to determine further testing strategy.

|  |
| --- |

Positive

Refer to reference laboratory (Public Health England, Colindale, UK) for further testing.

|  |
| --- |

* Enterobacter isolates positive for AmpC and showing only resistance to Ertapenem were not routinely referred. Stenotrophomonas maltophilia, and other intrinsically carbapenem resistant organisms, were excluded once identified.

Refer to duty microbiologist for discussion of further testing, reference laboratory confirmation and interpretation.* All E. coli and Klebsiella sp. with confirmed carbapenem resistance referred for confirmation.

***Infection control interventions***

**Table S1.** **Infection control interventions before, during and after the LTH KPC outbreak**

| Infection control theme | Pre-outbreak infection control interventions | Post-outbreak infection control interventions |
| --- | --- | --- |
| Hand hygiene | Infection control team hand hygiene audit results with 100% compliance  25/10/13 (prior to outbreak detection). | Infection control team audits throughout outbreak period |
|  | Peer-led audits (results not available) | Peer-led audits |
|  | - | Volunteers supervised hand hygiene on arrival/departure from the ward |
|  | - | Two additional mobile wash stations used on the ward during the outbreak (October 2013-January 2014) |
| Cohorting and isolation | Nursing staff cared for patients throughout the unit | Nursing staff initially dedicated to KPC-positive or negative patients only (not sustained for the entire outbreak due to staffing availability) |
|  | Gloves and aprons used for patients in source isolation (no sleeves) | Patients in source isolation were managed using dedicated equipment and single use long-sleeve gowns |
|  | Infection control nurse input as required for specific infections (e.g. *C. difficile*, MRSA) | Daily input from infection control nurse |
|  | Bed base 32 | Bed base reduced to 24 to facilitate effective cohorting |
|  | Students and volunteers regularly attending the ward | All non-essential staff excluded from ward with volunteer staff monitoring entry and exit to the ward |
|  | Patients not cohorted pending CPE results unless ‘high-risk’ (i.e. previous CPE positive patient or from hospital with known CPE) | All patients cohorted pending screening results |
|  | Glove and gown audits (results not available) | Glove and gown audits |
| CPE screening | Rectal CPE screening for all patients on admission to the unit | Rectal CPE screening for all patients on admission and discharge from the liver unit (including to/from other wards), and weekly whilst inpatients |
|  | Patients requiring transplant assessment admitted to the liver unit. | All referrals to the unit were triaged by a consultant hepatologist/surgeon and, where possible, patients requiring transplant assessment were seen in the outpatient department |
|  | Regular transfer of patients from geographical areas with recognised CPE problem | Transfer of inpatients from areas with endemic CPE was interrupted for a short period |
| Environmental cleaning | Normal cleaning activities | Education and support to enhance normal cleaning activities on the ward |
|  | Hydrogen peroxide vaporisation had not been employed on the unit prior to the outbreak | Environmental cleaning occurred including hydrogen peroxide vaporisation of the entire liver unit on 4/11/13 and 18/11/2013 (the ward was fully decanted for the second vaporisation) |
| Education | Weekly anti-microbial stewardship ward rounds with a consultant microbiologist | Daily anti-microbial stewardship was maintained by regular input from a consultant microbiologist, including out-of-hours support to minimise carbapenem prescribing |
|  | General infection control advice (posters) displayed on the ward. Mandatory infection control training for all nursing staff and doctors | Written and verbal education for staff, patients and visitors was central to the infection control strategy |
|  |  | This included face-to-face educational sessions for all affected patients led by the infection control team |

***WGS data processing and analysis***

The reference sequences for mapping short Illumina reads in the mapping pipeline were either downloaded from NCBI (CP000647.1 for *Klebsiella pneumoniae*, NC_018106.1 for *Klebsiella oxytoca*, CP001918.1 for *Enterobacter cloacae*, JH414876.1 for *Citrobacter freundii*) or generated within this study from long-read data (case 5; chromosomal hybridSPAdes assembly) for the dominant KPC-*Kpn-*ST661 strain (see below).

Consensus fasta sequences from the pipeline were used to reconstruct phylogenetic trees for each species with IQTree,^1^ using a GTR+G model and a maximum parsimony starting tree. The phylogeny was corrected for recombination using ClonalFrameML with default parameters.^2^ Short-read sequences were also assembled using SPAdes^3^ (Version 3.6) and the assemblies used for *in silico* multi-locus sequence-typing (MLST), plasmid Inc typing and Tn*4401* typing by BLASTn. For plasmid typing, we downloaded all publically available complete plasmids from NCBI (query term: plasmids AND Enterobacteriaceae AND complete sequence), de-duplicated them, and extracted any carrying *bla*_KPC_ alleles. Additional fully closed plasmid sequences were obtained from a global KPC study.^4^ These plasmid sequences were then used as references to identify similar *bla*_KPC_-carrying plasmid structures in the outbreak using BLASTn and local reassembly using SPAdes.

MinION sequencing data were processed by poretools^5^ to extract 2D reads. Multiple approaches to generate assemblies were applied using both MinION long-read and Illumina short-read data, namely hybridSPAdes,^3^ npScarf^6^ and Canu.^7^ HybridSPAdes combines Illumina and MinION reads to improve assemblies. npScarf builds on an Illumina only SPAdes assembly by using the long-read data to scaffold the initial assembly. Canu only uses MinION reads for assembly and is more prone to nucleotide-level error; Canu assemblies were therefore “polished” with Illumina short-read data using pilon.^8^ The various assemblies were then compared using dnadiff/mummerplot^9^ to identify discrepancies. Assembly integrity was checked using REAPR,^10^ assembly discrepancies were manually inspected to obtain fully circularised *bla*_KPC_ plasmid structures where possible. A plasmid was defined as circularised if it had >100bp overlapping ends with 100% sequence identity for hybridSPAdes/npScarf assemblies, and >1kbp overlapping ends at >99% sequence identity for Canu assemblies.

Resistance mechanisms were identified using resistType (https://github.com/hangphan/resistType), a method combining both assembly/BLASTn and mapping of sequencing reads to a panel of known reference mechanisms, including both chromosomal and acquired mechanisms.

**MinION sequencing methods and sequencing results**

*DNA extraction and sequencing*

Isolates were cultured from frozen stocks (-80°C) on Colombia Blood agar in the presence of three ertepenem discs (10µg; Oxoid, Thermo Fisher Scientific, USA) overnight at 37°C. Genomic DNA was isolated using the Qiagen Genomic-tip 100/G kit (Qiagen, Germany) following the manufacturer’s recommendations. DNA was quantified using the Qubit 2.0 Fluorometer (Life Technologies, USA) and fragment length assessed using the TapeStation 2200 (Agilent, UK).

*MinION sequencing*

DNA was prepared for sequencing using a modified version of the manufacturer’s protocol (SQK-NSK007; ONT, UK). Fragmentation was not performed and library preparation began with 4-6 µg of DNA. All bead-based clean-up steps were extended to 10 minutes binding incubation and 10 minutes elution at 37°C. All other steps were performed following the manufacturer’s protocol. Libraries were sequenced using the best available flow cells at the time (3/5 samples sequenced using R9 FLO-MIN104 flow cells, and 2/5 sequenced using R9 spot-on FLO-MIN105 flow cells). Libraries were topped-up after 12-24 hours, and sequencing performed for 48 hours in total. Data were base-called in real-time via Metrichor, using the best available workflow at the time (ONT, UK).

*MinION sequencing data – summary statistics*

MinION sequencing coverage of the five chosen isolates was 26~43x (Table S3), with sufficient long reads (489~5,435 >10kb) to enable the assembly of Tn*4401* (~10kb)-carrying plasmids in all cases. *C.freundii* (case 12) had the lowest coverage (26x) with the smallest number of >10kb long reads (489 reads); Case 12’s Kpne-ST250 had the highest coverage (43x) and case 18 (Kpne-ST321) the most reads >10kb (5,435 reads).

|  | Isolate name | | | | |
| --- | --- | --- | --- | --- | --- |
|  | Case 5 | Case 12_1 | Case 18 | Case 16 | Case12_2 |
| Sequence type  Total bases | Kpne-ST661  149,193,024 | *C.freundii*  143,098,651 | Kpne-ST321  208,100,861 | Kpne-ST1820  162,228,982 | Kpne-ST250  236,418,256 |
| Estimated coverage | 27.13 | 26.02 | 37.84 | 29.50 | 42.99 |
| Number of 2D reads | 37,825 | 70,298 | 35,874 | 31124 | 70,723 |
| Mean read length (bp) | 3,944 | 2,036 | 5,801 | 5,212 | 3,343 |
| Maximum read length (bp) | 95,222 | 57,214 | 61,358 | 68,750 | 65,198 |
| Minimum read length (bp) | 112 | 107 | 114 | 120 | 126 |
| Number of reads >5kb | 8,918 | 4,185 | 14,468 | 9,922 | 11,177 |
| #Reads>10k | 1,997 | 489 | 5,435 | 4,183 | 3,194 |
| #Reads<1k | 2,483 | 17,041 | 788 | 3,402 | 7,337 |

**Table S2: MinION long-read sequencing data summary**

***Mis-assembly by hybridSPAdes for case 16***

For case 16, *bla*_KPC_/Tn*4401* was found on a 5.5Mb chromosomal contig in the hybridSPAdes assembly, a 100kb contig in the npScarf scaffolded assembly, and a truncated Tn*4401* (not including *bla*_KPC_) on a 177kb contig in the Canu assembly (Table S3). The truncation of Tn*4401* and absence of *bla*_KPC_ in the Canu assembly was an assembly error, confirmed by mapping and visualising read pileups with respect to the hybridSPAdes chromosomal contig, and using REAPR. The hybridSPAdes assembly, on the other hand showed an integration of an IncF plasmid into the chromosomal contig (generating a contig of ~5.5Mb in size). There was a 13kb repeat structure at position 1bp and 5.364Mb on this 5.5Mb contig. The mapping of MinION long-reads to this contig showed two-fold sequence coverage at this repeat structure (Figure S1). This indicated a potential mis-assembly error at the 5.346 Mb breakpoint. When the long 5.5Mb contig was split into two smaller contigs, the 5.364Mb subcontig shared 92% sequence similarity with the CP012252.1 *K. pneumoniae* complete genome and the 187kb plasmid subcontig shared 83% sequence similarity with a pKPN_262 (CP007734.1) complete plasmid.

**Figure S1. Long-read sequence alignment against the hybridSPAdes assembly at putative boundaries between plasmid and chromosomal context (mid-point coordination 5.364Mbp) for isolate in case 16**

***
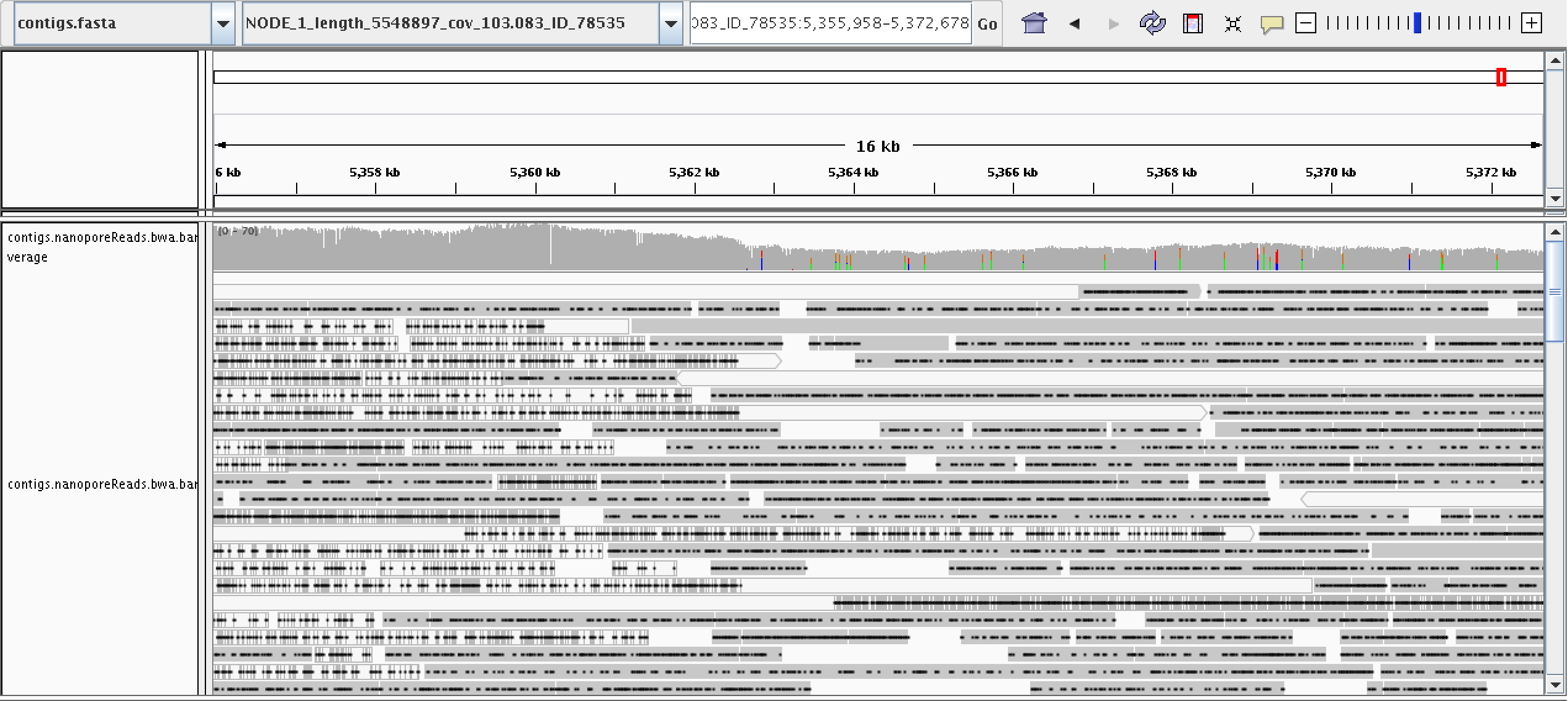
***

***Maximum likelihood phylogeny inferred by ClonalFrameML***

**Figure S2. Phylogenetic tree of *K.pneumoniae* ST-661 isolates estimated by IQTREE and corrected for recombination using ClonalFrameML.**

******

***SCOTTI analysis***

*Outbreak analysis*

We applied SCOTTI,^11^ a structured coalescent-based tool for reconstructing transmission within outbreaks, to the dominant outbreak KPC-*Kpn* ST661 isolates, combining epidemiological and chromosomal genomic data. We masked the recombinant regions detected by ClonalFrameML, and used the resulting genome alignment as input to SCOTTI, together with the first date where a KPC-E isolate was detected in a patient, and the start and end date of each patient’s infection risk period (see below). We used a **π** prior distribution for the mutation rate (mean 2E-6^12^) and a uniform prior distribution between 14-16 for the number of hosts (also allowing possible non-sampled hosts).

*Definition of start and end date of infection risk period*

The start date of the infection risk period was defined as:

- The last date of a negative KPC screen before the first KPC detection (where information is available)
- Or the start date of the most recent period of stay (in the liver unit where applicable) before the first KPC detection where possible
- Or the start date of the period of stay when KPC was first detected if there were no other previous admissions.

The end date of the infection risk period is defined as:

- The first date of a negative screening result of a patient after the first KPC detection date (where information is available)
- Or the end date of the last admission period.

The SCOTTI model requires the first KPC detection date to lie between the start and end date of the infection risk period. However, for Case 7 from outside the Leeds region, the first KPC detection date was later than the admission period to LTH. As the patient had not been admitted to any other hospital trust between last discharge from LTH and KPC detection, and the WGS data for this case were highly similar to other outbreak strains (both chromosomal sequences and plasmid typing results), this case was included in the outbreak. Because the infection risk period was only limited to the admission period, the first KPC detection date for this case was re-assigned to the last date of the admission period for the SCOTTI analysis only.

Table S3. Summary assembly statistics of different assembly approaches combining long MinION and short Illumina sequencing reads

| Sample name | Assembly method | Total bases | Number of contigs | Mean contig length | Maximum contig length | Minimum contig length | Tn*440*1 contig | Tn*4401* contig length | Tn*4401* plasmid closure status |
| --- | --- | --- | --- | --- | --- | --- | --- | --- | --- |
| Case 5 | hybridSpades | 5,738,527 | 10 | 573,853 | 5,266,292 | 732 | NODE_3_length_116898_cov_243 | 116,898 | closed, 5kb overlapping ends |
|  | npScarf | 5,599,304 | 7 | 799,901 | 5,264,539 | 4,350 | Scaffold36 | 43,168 | no |
|  | Canu | 5,708,605 | 9 | 634,290 | 3,236,665 | 10,403 | tig00000005_pilon | 115,067 | no |
|  | hybridSpades | 5,696,699 | 23 | 2,476,823 | 2,066,639 | 670 | NODE_10_length_69021_cov_284 | 69,021 | no |
| Case 12_1 | npScarf | 5,585,466 | 10 | 558,547 | 4,380,112 | 2,747 | Scaffold32 | 90,677 | no |
|  | Canu | 5,759,687 | 16 | 359,980 | 2,069,264 | 5,077 | tig00000004_pilon | 104,038 | closed, 4.7kb overlapping ends |
|  | hybridSpades | 5,714,247 | 2 | 2,857,123 | 5,406,273 | 307,974 | NODE_2_length_307974_cov_117 | 307,974 | no |
| Case 18 | npScarf | 5,621502 | 4 | 1,405,375 | 5,391,467 | 6,624 | Scaffold23 | 203,997 | no |
|  | Canu | 5,772,040 | 4 | 1,443,010 | 5,414,878 | 22,895 | tig00000001_pilon | 121,973 | closed, 15kb overlapping ends |
|  | hybridSpades | 5,698,011 | 8 | 712,251 | 5,548,897 | 555 | NODE_1_length_5548897_cov_103 | 5,548,897 | No, probably misassembly |
| Case 17 | npScarf | 5,552,361 | 5 | 1,110,472 | 5,327,502 | 14,036 | Scaffold50 | 10,0860 | no |
|  | Canu | 5,618,639 | 5 | 1,123,727 | 5,290,580 | 6,461 | tig00000006_pilon | 176,824 | no |
|  | hybridSpades | 6,275,925 | 11 | 570,539 | 5,532,197 | 1,968 | NODE_5_length_68647_cov_216 | 68,647 | no |
| Case 12_2 | npScarf | 6,199,578 | 7 | 885,654 | 5,525,663 | 1,920 | Scaffold45 | 68,853 | no |
|  | Canu | 6,233,271 | 11 | 566,661 | 4,039,509 | 3,758 | tig00000011_pilon | 114,156 | closed, 15kb overlapping ends |

**References**

1. Nguyen LT, Schmidt HA, von Haeseler A et al. IQ-TREE: a fast and effective stochastic algorithm for estimating maximum-likelihood phylogenies. *Mol Biol Evol* 2015; **32**: 268-74.

2. Didelot X, Wilson DJ. ClonalFrameML: efficient inference of recombination in whole bacterial genomes. *PLoS Comput Biol* 2015; **11**: e1004041.

3. Bankevich A, Nurk S, Antipov D et al. SPAdes: a new genome assembly algorithm and its applications to single-cell sequencing. *J Comput Biol* 2012; **19**: 455-77.

4. Stoesser N SA, Peirano G, Anson LW, Pankhurst L, Sebra R, Phan HTT, Kasarskis A, Mathers AJ, Peto TEA, Bradford P, Motyl MR, Walker AS, Crook DW, Pitout JD. Genomic epidemiology of global Klebsiella pneumoniae carbapenemase (KPC)-producing Escherichia coli *in submission* 2016.

5. Loman NJ, Quinlan AR. Poretools: a toolkit for analyzing nanopore sequence data. *Bioinformatics* 2014; **30**: 3399-401.

6. Cao MD, Nguyen SH, Ganesamoorthy D et al. Scaffolding and Completing Genome Assemblies in Real-time with Nanopore Sequencing. *bioRxiv* 2016.

7. Berlin K, Koren S, Chin CS et al. Assembling large genomes with single-molecule sequencing and locality-sensitive hashing. *Nat Biotechnol* 2015; **33**: 623-30.

8. Walker BJ, Abeel T, Shea T et al. Pilon: an integrated tool for comprehensive microbial variant detection and genome assembly improvement. *PLoS One* 2014; **9**: e112963.

9. Kurtz S, Phillippy A, Delcher AL et al. Versatile and open software for comparing large genomes. *Genome Biol* 2004; **5**: R12.

10. Hunt M, Kikuchi T, Sanders M et al. REAPR: a universal tool for genome assembly evaluation. *Genome Biol* 2013; **14**: R47.

11. De Maio N, Wu CH, Wilson DJ. SCOTTI: Efficient Reconstruction of Transmission within Outbreaks with the Structured Coalescent. *PLoS Comput Biol* 2016; **12**: e1005130.

12. Stoesser N, Sheppard AE, Pankhurst L et al. Evolutionary History of the Global Emergence of the Escherichia coli Epidemic Clone ST131. *MBio* 2016; **7**: e02162.
